# Heterogeneity of tumor-infiltrating lymphocytes ascribed to local immune status rather than neoantigens by multi-omics analysis of glioblastoma multiforme

**DOI:** 10.1038/s41598-017-05538-z

**Published:** 2017-07-31

**Authors:** Lin Feng, Haipeng Qian, Xuexin Yu, Kan Liu, Ting Xiao, Chengli Zhang, Manchao Kuang, Shujun Cheng, Xueji Li, Jinghai Wan, Kaitai Zhang

**Affiliations:** 1 National Cancer Center/Cancer Hospital, Chinese Academy of Medical Sciences and Peking Union Medical College, Beijing, 100021 China; 2 Department of Neurosurgery, National Cancer Center/Cancer Hospital, Chinese Academy of Medical Sciences and Peking Union Medical College, Beijing, 100021 China; 3 College of Bioinformatics Science and Technology, Harbin Medical University, Harbin, 150008 China; 4 Department of Diagnostic Radiology, National Cancer Center/Cancer Hospital, Chinese Academy of Medical Sciences and Peking Union Medical College, Beijing, 100021 China

## Abstract

Hypothetically, intratumoral genomic heterogeneity has the potential to foster tumor-infiltrating lymphocyte (TIL) diversity; however, no study has directly tested this hypothesis by simultaneously investigating somatic mutations, TIL diversity, and immune response activity. Thus, we performed whole-exome sequencing, immune repertoire sequencing and gene expression on ten spatially separated tumor samples obtained from two tumor masses excised from a glioblastoma multiforme (GBM) patient, and we included peripheral blood as control. We found that although the multi-region samples from one tumor shared more common mutations than those from different tumors, the TIL populations did not. TIL repertoire diversity did not significantly correlate with the number of non-synonymous mutations; however, TIL diversity was highly correlated with local immune activity, as the pathways were all immune-related pathways that highly positive correlated with local TIL diversity. Twenty-three genes with expression largely unaffected by the intratumor heterogeneity were extracted from these pathways. Fifty GBM patients were stratified into two clusters by the expression of these genes with significant difference in prognosis. This finding was validated by The Cancer Genome Atlas (TCGA) GBM dataset, which indicated that despite the heterogeneity of intra-tumor immune status, the overall level of the immune response in GBM could be connected with prognosis.

## Introduction

GliobGlastoma multiforme (GBM) is the most aggressive subtype of adult brain cancer, and the median survival of GBM patients is typically less than 2 years^1–3^. As suggested by “multiforme”, GBM shows significant intratumoral heterogeneity at the cellular and molecular levels^4, 5^. In the past decade, the extensive genomic inter-^6, 7^ and intra-tumoral^8^ heterogeneity of GBM tumors has been revealed. However, the intra-tumor heterogeneity of immune status in GBM remains unexplored. The genomic heterogeneity in tumor cells likely triggers different *in situ* immune responses, and it might be driven by the selection on immunity^9^. The relationship between the heterogeneity of somatic mutations and local immune status warrants investigation.

The generation of immunity to cancer is a self-propagating cyclic process starting with the release of antigens from cancer cells and ending with the infiltration of activated effector T cells into the tumor bed (so-called TILs) and the killing of their targets^10^. This adaptive immune response to tumors has re-emerged as a favorable prognostic marker for various cancers^11^. Considering the prognostic information added by the presence of TILs, an international consortium has been initiated to implement the pathology methodology named ‘Immunoscore’ (which is used to quantify the *in situ* TILs) into standard cancer classification methods^12^. However, the role of the presence of TILs in GBM remains contentious^13^, as some research supports an association between TILs and the prolonged survival of GBM patients^14^ and other research does not^15^. The introduction of a new technology may provide insight into this issue.

The repertoire of antigen-specific αβ-T cells is generated by somatic recombination of the T cell receptor α- and β-chains. Rearrangement of the T cell receptor beta (TCRB) locus generates the highly variable complementary determining region 3 (CDR3), which is critical in determining the specificity of each particular T cell clone. NGS technology can be used to sequence tens of thousands of CDR3 region sequences in parallel. This technology, generally referred to as TCR sequencing or immune repertoire sequencing, has been widely used and has provided insight into the dynamic patterns of T cell subsets from peripheral blood^16, 17^, the clonal composition of autoreactive T cells in autoimmune diseases^18^, and even the spatial heterogeneity of TILs^9, 19, 20^. As mentioned by Chen *et al*.^20^, reported findings on the spatial heterogeneity of TILs have been inconsistent. One hypothesis is that heterogeneous intratumoral T cell immune responses can be activated by heterogeneous local neoantigens associated with the tumor^20^. Here, we assess the clonal composition of TILs, the somatic mutations of tumor cells, and the local immune context simultaneously using advanced high-throughput technologies.

To depict an overall view of the intra-tumoral heterogeneity by referring to cancer cells and their immune context and then analyze the complex relationships between the TILs and cancer cells, TCR sequencing, whole-exome sequencing, and gene expression profiling analysis were performed simultaneously on ten spatially separated tumor samples obtained from two GBM masses (the primary tumor and its associated metastatic lesion) from a patient (hereafter referred to as “Patient 1”). We found that TIL heterogeneity was not related to the presence of protein-altered mutations but was coordinated to the local immune response activity.

## Materials and Methods

**Figure 1:**
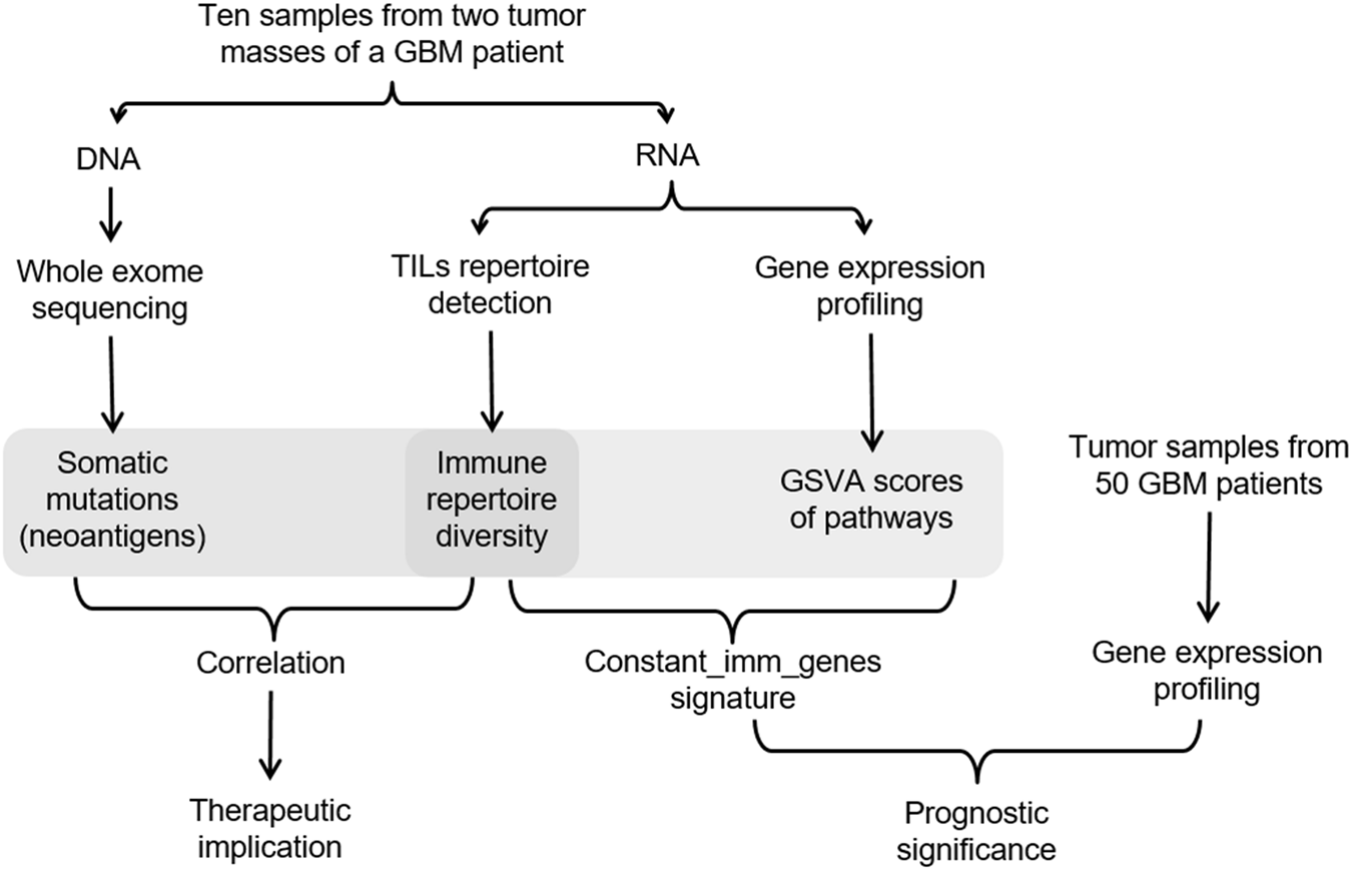
Schematic diagram of the study design.

A flowchart of the study design is shown in Fig. 1.

### Patients and tumor samples

**Figure 2:**
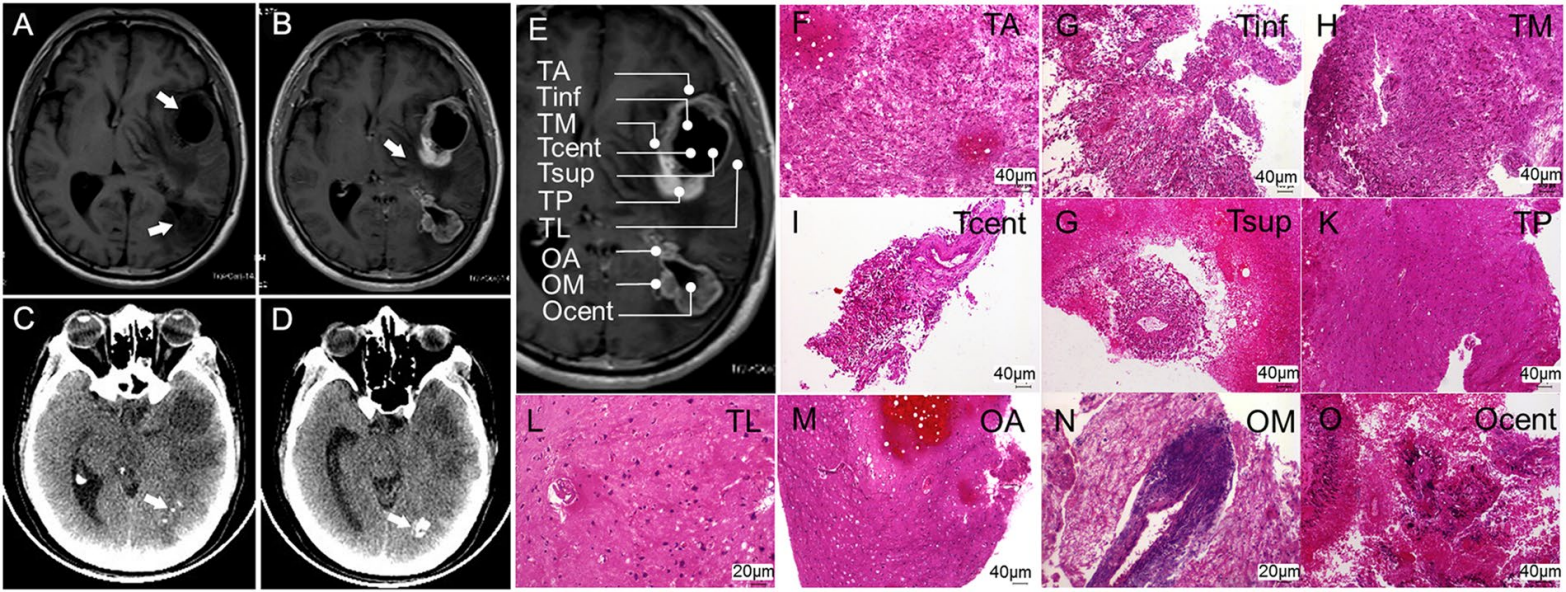
Clinical imaging and morphological features of the tumors (biopsies) of Patient 1. Two low-signal-intensity areas in the left temporal and occipital lobes and the ring-like peripheral edema are shown in non-enhanced (**A**) and corresponding enhanced T1-weighted MRI images (**B**). The lesions and edema are indicated by arrows. Calcification (indicated by arrows) was apparent in the occipital lesion under non-enhanced CT (**C** and **D**). (**E**) The sites of ten biopsies harvested from the surgical resection specimen are represented schematically on the MRI image. (**F**–**O**) Histologic specimens of the ten tumor samples stained with H&E.

Patient 1, who had two brain neoplasms (Fig. 2A–D), underwent surgery on Dec 28, 2012, in the Department of Neurosurgery at the National Cancer Center/Hospital of the Chinese Academy of Medical Sciences. Samples (three from the occipital tumor and seven from the temporal tumor) were harvested from the surgically resected specimen to represent the spatial extent and macroscopic heterogeneity (Fig. 2E). Four milliliters of blood was drawn into a K2EDTA tube (BD, USA) before the surgery. An additional 50 samples that corresponded to the microscopic sections containing >50% tumor cells and that were obtained from patients treated in the same department between September 2005 and June 2009 were used for the expression profiling analysis. The clinical features of all of the patients are presented in Table S1. The use of all of the human tissue samples and the experimental procedures for this study were reviewed and approved by the Ethics Committee of the National Cancer Center/Hospital of the Chinese Academy of Medical Sciences, and all of the patients provided written informed consent. All the methods were carried out in accordance with the approved guidelines.

### Nucleic acid purification

The genomic DNA and total RNA were purified simultaneously from each of the ten samples of Patient 1 using the AllPrep DNA/RNA mini kit (Qiagen, USA). The germline DNA was extracted from peripheral blood mononuclear cells of Patient 1 using the DNeasy Blood & Tissue kit (Qiagen, USA). Total RNA from the other 50 GBM samples and from the peripheral blood mononuclear cells of Patient 1 was isolated using TRIzol reagent (ThermoFisher, USA) and purified using the RNeasy kit (Qiagen, USA). All of the DNA and RNA used in this study exhibited OD 260/280 ratios above 1.9, and all of the RNA samples displayed integrity numbers greater than 6.5.

### TCR sequencing

The TCR sequences were obtained via a series of three nested PCR reactions using the primers as described in ref. 21 (Table S2) with a slightly modified protocol of the ARM-PCR procedure^16, 22^. TCRB-specific cDNA was synthesized from 0.5 μg of total RNA using ProtoScript II Reverse Transcriptase (NEB, UK) and primer TRBCRo. For the first PCR step, cDNA templates were pre-amplified using a multiplex PCR 5x Master Mix (NEB, UK) with multiple Vβ region primers (TRBV1Fo-TRBV30Fo) and a Cβ region primer (TRBCRo primer). The PCR protocol was 95 °C for 3 min, 10 cycles of 95 °C for 30 sec, 60 °C for 2 min, and 68 °C for 1 min; and then 68 °C for 5 min. Two microliters of the PCR product, the TRBV1Fi-TRBV30Fi, TRBCRi primer and H2O were added to the NEB multiplex PCR master mix for the second round of PCR without changing the cycling conditions. Two microliters of the second PCR product was used as a template for the third PCR reaction, which incorporated barcodes and sequencing adapters into the final product. At this step, amplification was performed using Deep Vent_R_ (exo-) DNA polymerase (NEB, UK) for 25 cycles using primers SuperF and SuperR. The final product was run on a 1.2% agarose gel. The band of approximately 250 to 500 bp was excised and gel-purified using a QIAquick gel extraction kit (Qiagen, USA) and then sequenced on the illumina HiSeq 2500 platform (pair-end 250). The raw data of TCR sequencing are available from the SRA database (accession number SRP076444).

### TCR sequencing data analysis

The raw data were cleaned using Trimmomatic^23^. Then, we used FLASH^24^ to merge the paired reads to obtain the complete sequence of the CDR3 regions. To assign the rearranged mRNA sequences to their germline V, D, and J counterparts, we used MiGEC^25^. We used VDJtools^26^ to accomplish the basic statistics and diversity analysis (calculation of the Shannon-Wiener diversity index (hereafter, ShannonDI)) of the TCR clones.

### Multi-region exome sequencing

All samples from Patient 1 were normalized to 1 μg DNA and sheared to 150–200 bp. The DNA fragments were enriched with Agilent SureSelect Human All Exon V6 kits and end-repaired and adapter-ligated using the Paired-End DNA Sample Prep Kit (Illumina, USA). The capture libraries passing the quality control procedure were sequenced using Illumina HiSeq 2000 (pair-end 100). The raw sequencing data are available from the SRA database (accession number SRP076444).

### Somatic mutation calling and validation

First, the adapter sequence in the raw data was removed, and the low quality reads were discarded. The cleaned reads were aligned to hg19 using the Burrows-Wheeler Aligner (BWA)^27^. The aligned sam files were converted to bam format and sorted, and PCR duplications were removed using Picard tools (broadinstitute.github.io/picard/). Using the typical GATK^28^ workflow, the data were processed for local InDel realignment and base quality recalibration. VarScan^29^ was used for somatic mutation calling. The calls were further filtered as follows: a minimum of 10× coverage in the germline sample with zero non-reference reads, the variant annotated as protein-altered by ANNOVAR^30^, and multiple variants located in a genomic interval longer than 100 bp within the identical site. The filtered somatic mutations were validated and verified using Sanger sequencing.

### mRNA expression analysis

The mRNA expression profiles of tumor samples from Patient 1 and the 50 additional GBM patients were generated using Agilent 4 × 44 K Whole Human Genome Oligo Microarrays (Agilent, USA). The data were normalized using the quantile method of the “limma”^31^ R package. All of the raw and processed data are available from the GEO database (accession numbers GSE83300 and GSE83301). The raw mRNA expression data and the corresponding clinical information, which was available for 487 GBM samples, were downloaded from the Cancer Genome Atlas (TCGA) database (https://tcga-data.nci.nih.gov/tcga/tcgaHome2.jsp) and processed using the RMA methodology included in the “affy”^32^ R package.

### Additional statistical analysis

The Gene Set Variation Analysis^33^ (GSVA, from the R “GSVA” package) program was applied to the expression profiling of the Patient 1 samples. Spearman’s correlation and the Wilcoxon rank-sum test were performed using the R package “stat”. The survival curves were determined by the Kaplan-Meier method and examined by the log-rank test with the R package “survival”.

## Results

### Clinical imaging and tumor morphology of Patient 1

Two low-signal-intensity areas in the left temporal and occipital lobes were identified by non-enhanced T1-weighted MRI before surgery (Fig. 2A). A ring-like enhanced lesion and a peripheral enhanced lesion with peripheral edemas were identified by the corresponding enhanced T1-weighted MRI (Fig. 2B). Both lesions were hypo-dense, and the occipital lobe lesion bore patchy and lumpy calcifications as observed on the non-enhanced CT (Fig. 2C and D). This finding indicated a multifocal high-grade brain tumor wherein the tumor in the occipital lobe might have appeared earlier considering the calcification. Ten samples, hereafter referred to as OA, Ocent, OM, TA, Tcent, Tinf, TL, TM, TP and Tsup (Fig. 2E), were harvested from the surgically resected specimen of the occipital (the first three) and temporal (the remaining seven) tumor masses. All of the samples were subjected to pathological examination by H&E staining. As shown in Fig. 2F–O, significant variety in morphology was observed among the ten samples. In particular, perivascular lymphocytic cuffing of blood vessels was observed in OM (Fig. 2M), Ocent featured pseudopalisading necrosis and multi-nucleated giant tumor cells (Fig. 2O), and six out of seven temporal tumor samples (TA, Tinf, TM, Tcent, Tsup and TP) appeared morphologically similar by microscopy and were characterized by the proliferation of glial cells with elongated, fusiform nuclei accompanied by microvascular proliferation and necrosis (Fig. 2F–K). Almost no tumor cells were observed in OA and TL (Fig. 2L,M).

### High-throughput sequencing revealed distribution characteristics of TILs and circulating T cells repertoires in Patient 1

The TCRB CDR3 regions of the TILs were sequenced in parallel. On average, 3.73 million TCRB sequences, distributed among an average of 94,617 unique TCRB CDR3 rearrangements, were obtained for each tumor sample (Table S3). Identical analyses were performed on the peripheral blood of Patient 1 (Table S3).

**Figure 3:**
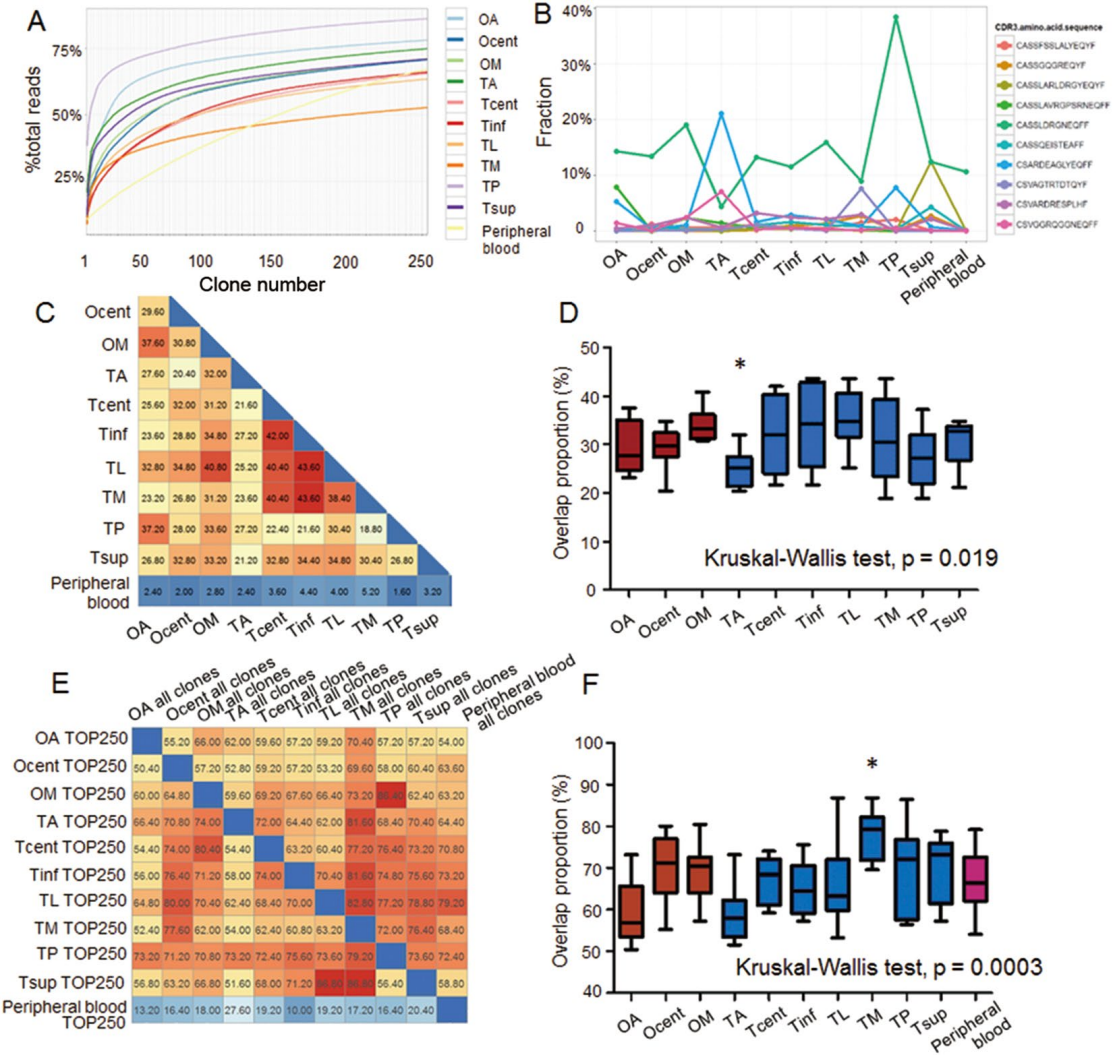
Distribution characteristics and heterogeneity of the TCR repertoires of Patient 1. (**A**) Cumulative size of the TOP250. The x-axis depicts the number of clones included (always starting from the most expanded clones). The y-axis shows the percentage of TCRB sequences that are covered by the included clones. (**B**) Frequencies of the ten most abundant common TCRB clones. The y-axis shows the percentage of the corresponding clone. The pairwise overlaps of the TOP250 among all samples are shown in (**C**). The boxplot of the overlaps of each sample with other samples according to the results from (**C**) is shown in (**D**). The pairwise overlaps of the TOP250 in one sample with the entire repertoire of the other samples are displayed in (**E**). The boxplot of the overlaps of each sample according to the results from (**E**) is shown in (**F**). Significant differences are marked by an asterisk (*).

The percentages of TCRB clones with different frequencies are summarized in Table S4. The TCR clones showed a right-skewed frequency distribution, i.e., a tiny fraction of highly expanded clones. A cumulative frequency graph (Fig. 3A) was generated for the top 250 most expanded clones (hereafter, TOP250) in each sample. The cumulative frequencies of the TOP250 for all samples were greater than 50% (range: 52–86%), indicating a domination of the TIL repertoire by a small fraction of clones. The cumulative frequencies of the top 30 clones were considerably higher than for other clones for tumor samples but not peripheral blood. The cumulative frequencies of the TOP250 for the peripheral blood showed an almost constant slope (Fig. 3A, yellow line), which indicated a more uniform TCRB clone frequency distribution in the peripheral blood than in the TILs. In summary, we observed fewer highly expanded αβ-T cell clones in the peripheral blood than in the TILs, which is consistent with a previous study^34^.

### Heterogeneity of the TCR repertoires of Patient 1

The TCRB rearrangements shared between different tumor samples and between tumor samples and the peripheral blood were identified. The average of the overlapping ratios between the multi-region tumor samples was 14.87%, whereas the overlapping ratios between the tumor samples and the peripheral blood was 6.06% (Figure S1). In total, 1,180 TCRB clones (~0.15% of all unique clones) were shared among all of the samples, of which the ten most frequent clones are shown in Fig. 3B. A common clone (Fig. 3B, green line) with the highest frequency was observed in all of the samples (including peripheral blood) except TA. The frequency of this clone in the peripheral blood was 10.6%, and the frequencies of the other nine top 10 clones ranged from 0.43–0.53%. The most frequent clone in TA (Fig. 3B, blue line) differed from that of the other sites.

As the TOP250 were dominant in all of the TCRB repertoires, the overlapping among samples was further investigated. First, the overlapping percentage of the TOP250 between each pairwise combination of the tumor samples and the peripheral blood was examined, as illustrated in Fig. 3C. The overlapping between the peripheral blood and the tumor samples was dramatically small (mean ± SE: 3.16 ± 1.14%). In contrast, the overlapping between tumor samples was approximately 20–40%. Statistical analysis revealed that TA demonstrated the lowest overlapping ratio with the other samples (Kruskal-Wallis test, p = 0.019, Fig. 3D), indicating a notable difference in the αβ-T cell repertoires between TA and the other samples. Second, the overlapping of the TOP250 in a certain sample with the entire TCRB repertoire for the other samples was examined. As shown by the rows in Fig. 3E, approximately 50–80% of the TOP250 in one tumor sample was present in the TCR repertoires of the other tumor samples and the peripheral blood. The total number of the TOP250 in the 10 tumor samples was 1,357. Of these highly expanded, tumor-related TCRB clones, 1,002 (73.84%) were detectable in the peripheral blood, which constituted 0.84% of the TCRB clone numbers in the peripheral blood but accounted for 20.34% of the entire TCRB repertoire in the peripheral blood. In comparison, the TOP250 in the peripheral blood were repeated in the TILs at a much lower frequency (17.76 ± 4.63%). As shown in Fig. 3F, with reference to the columns in Fig. 3E, TM, which demonstrated the largest overlapping rate, harbored the most compatible TIL repertoire (Kruskal-Wallis test, p = 0.0003).

These findings suggest that (1) enormous variety exists in the configuration of the TIL clones at all tumor sites (both within the same tumor mass and among separate tumor masses); (2) the number of highly expanded T cell clones in the peripheral blood is less than that observed in the TILs, among which the most highly expanded clones might be related to the tumor; and (3) most of the highly expanded αβ-T cell clones in the TILs were detectable in the peripheral blood, which occupied a considerable portion of the TCRB repertoire in the peripheral blood (~20%).

### Identification and validation of somatic mutational heterogeneity

**Figure 4:**
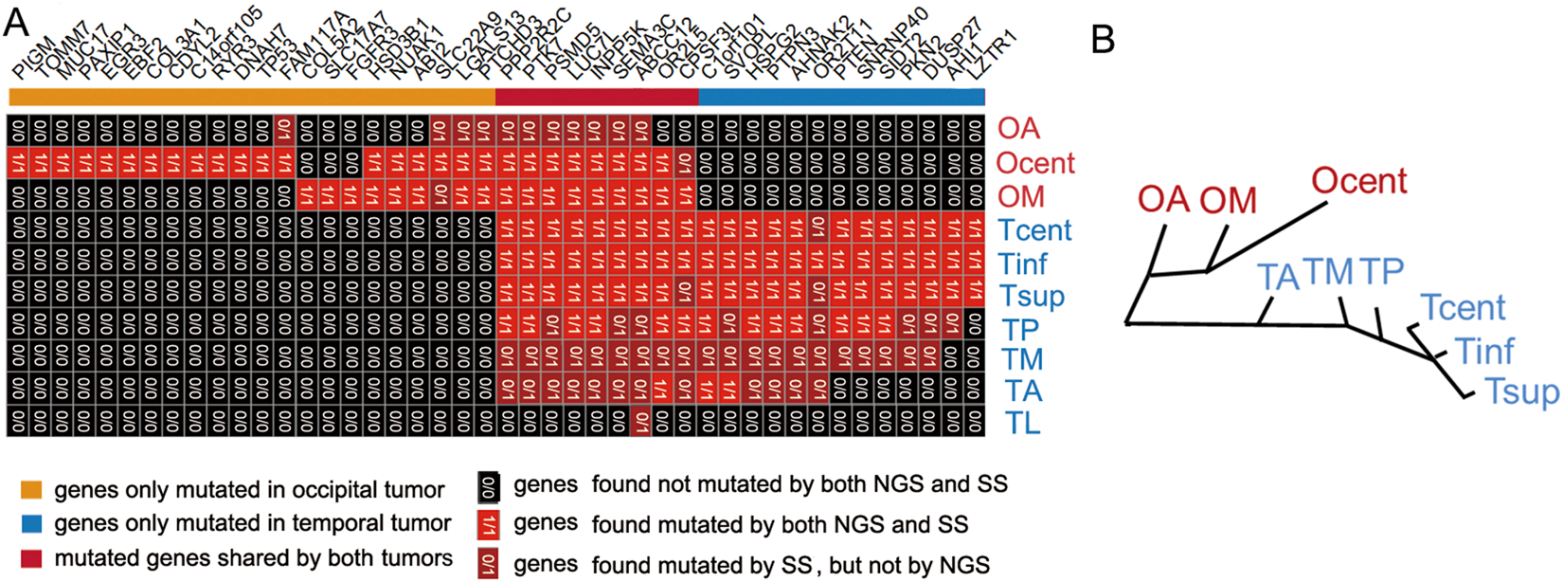
Genetic intra- and inter-tumor heterogeneity and phylogeny in Patient 1. (**A**) The regional distribution of 44 nonsynonymous point mutations in ten tumor samples. The heat map indicates the presence of a mutation (red/dark red) or its absence (black) in each region. The numbers in each cell indicate whether mutation presence was validated by NGS, Sanger sequencing (SS) or both. The color bars above the heat map indicate the classification of mutations according to whether they are unique or shared among regions. (**B**) shows phylogenetic relationships of the tumor regions; branch lengths are proportional to the number of nonsynonymous mutations separating the branching points.

Exon-capture sequencing was performed on the tumor samples and the peripheral blood mononuclear cells of Patient 1, resulting in a mean coverage of 105.3 (Table S5). The non-synonymous somatic mutations were filtered as described in Materials and Methods and manually reviewed. In total, 44 highly credible mutations were identified in the two tumors of Patient 1 (Fig. 4A and Table S6). Sanger sequencing (SS) validation showed a minimal false-positive rate of mutation calling; however, the false-negative rate was considerable (Figs 4A and S2). The false negative mutations missed by NGS were predominantly found in TM, OA and TA, which might have resulted from decreased tumor cellularity in these samples, an overly strict filtering criteria for mutations, or limited sequencing depth. Because the primary aim of our study was to analyze the tumor cells and their microenvironment, the sampling was performed regardless of the percentage of tumor cells.

Nine of the 44 identified mutations were shared by the two tumor masses (Fig. 4A), which suggested their homologous origin. Of the remaining 35 tumor-specific mutations, 22 occurred in the occipital tumor and 13 in the temporal tumor. We inferred ancestral relationships and constructed a phylogenetic tree of the separated tumor regions by the mutation profiles (Fig. 4B; TL was discarded due to a lack of detectable mutations), which revealed branching rather than linear tumor evolution. The heterogeneity of the occipital tumor samples was higher than that of the temporal tumor samples. Considering these results together with the imaging features (i.e., calcification and other features), the occipital tumor was inferred as the primary tumor, and the temporal tumor were inferred as its metastatic derivative.

### Relationship between TCR clone diversity and somatic mutations

**Figure 5:**
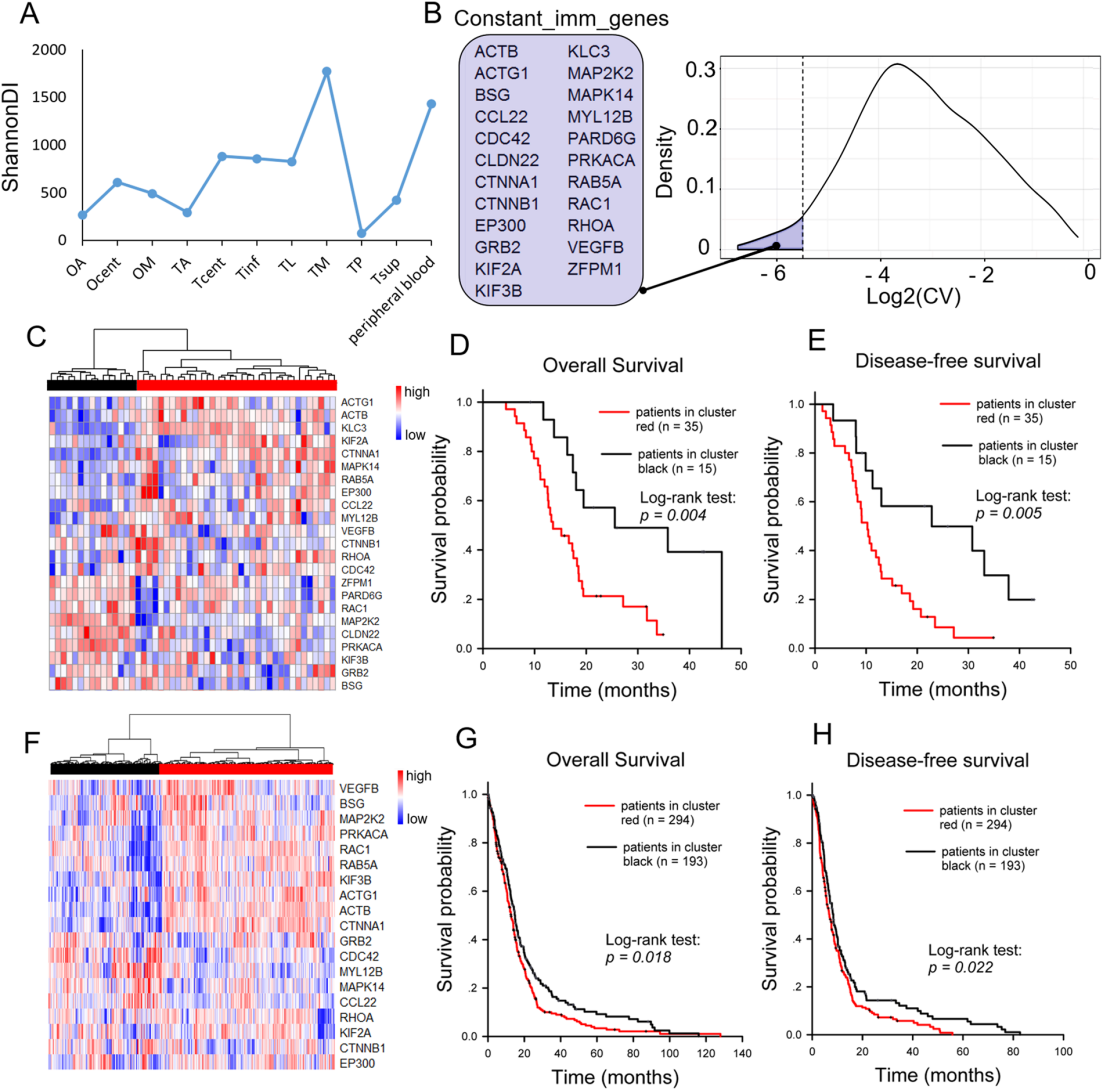
Discovery of the Constant_imm_genes and validation of their clinical prognostic significance. (**A**) ShannonDI of the samples. (**B**) The right panel shows the log2-transformed CV of the expression level of genes belonging to the pathways listed in Table 1. The dotted line indicates the selected cut-off threshold of −5.5, which defines the Constant_imm_genes listed in the left panel. (**C**) Fifty GBM patients were classified into two major groups (red and black clusters) by unsupervised hierarchical clustering according to the expression pattern of the Constant_imm_genes. The expression levels of these genes are illustrated as a color spectrum, with red, white and blue representing high, medium and low expression, respectively. Kaplan-Meier survival curves and log-rank tests were used to estimate the overall survival (**D**) and disease-free survival (**E**) of the two clusters of patients. A similar analysis was applied to the public TCGA GBM datasets, and the results are shown in panels (**F**–**H**).

The ShannonDI was applied to quantify the T cell clone diversity^35^. As shown in Fig. 5A, the ShannonDI fluctuated considerably in the 10 tumor samples. In particular, the highest TCRB diversity (in TM) was even higher than that of the peripheral blood, which had the expected highest TCRB diversity. This result indicated that the patient’s immunity might have been depressed systemically by the tumors. Although the tumor microenvironment harbored high heterogeneity, we observed no significant differences between the ShannonDIs of the occipital and temporal samples (Wilcoxon rank sum test, p = 0.5167), which suggested a similar immune microenvironment between the primary and metastatic tumors. Moreover, we observed no significant correlation between the ShannonDI and the number of non-synonymous mutations in the tumor samples (Spearman’s rank correlation, p = 0.4614).

### TCR clone diversity was highly correlated to immune pathway activities

The mRNA profiling of Patient1’s tumor samples was conducted using microarrays. To extract information reflecting the activity of biological pathways from the microarray data, 772 gene sets compiled from the pathway databases “KEGG”, “REACTOME” and “BIOCARTA” were downloaded from the Molecular Signatures Database v4.0^36^, and the GSVA scores^33^ of these gene sets were calculated for the ten tumor samples. The Spearman correlation analysis was applied to the GSVA scores and the ShannonDI of the tumor samples. Those gene sets with the GSVA scores that were highly positively correlated with the ShannonDI (Spearman correlation coefficient higher than 0.75) are listed in Table 1. The associated pathways were all immune-related pathways.

**Table 1:** The pathway-related gene terms of gene sets with GSVA scores that were highly positively correlated to the ShannonDI in the tumor samples.

Term	R*	p-value**
KEGG_LEUKOCYTE_TRANSENDOTHELIAL_MIGRATION	0.84	<0.01
KEGG_NATURAL_KILLER_CELL_MEDIATED_CYTOTOXICITY	0.84	<0.01
KEGG_CYTOKINE_CYTOKINE_RECEPTOR_INTERACTION	0.83	0.01
REACTOME_IMMUNOREGULATORY_INTERACTIONS_BETWEEN_A_LYMPHOID_AND_A_NON_LYMPHOID_CELL	0.81	0.01
REACTOME_INTEGRIN_CELL_SURFACE_INTERACTIONS	0.81	0.01
BIOCARTA_LYM_PATHWAY	0.77	0.01
REACTOME_FACTORS_INVOLVED_IN_MEGAKARYOCYTE_DEVELOPMENT_AND_PLATELET_PRODUCTION	0.77	0.01
REACTOME_CELL_CELL_JUNCTION_ORGANIZATION	0.77	0.01
KEGG_HEMATOPOIETIC_CELL_LINEAGE	0.77	0.01
BIOCARTA_IL17_PATHWAY	0.76	0.02
BIOCARTA_GRANULOCYTES_PATHWAY	0.76	0.02

*R: Spearman correlation coefficient.

**p-value: Spearman correlation analysis.

### The steadily expressed genes from TCR-diversity-correlated pathways were prognostically informative among GBM patients

There are 718 array-detectable genes in the 11 gene sets in Table 1. To find the steadiest among these genes, i.e., those least affected by the intratumor heterogeneity, we calculated the coefficient of variation (CV) of the expression levels in the ten tumor samples. The distribution of the log2-transformed CV values is shown in Fig. 5B; a cut-off threshold of −5.5 was selected at the inflection point of the curve, as indicated by the dotted line. The 23 genes that crossed the cut-off threshold (the marked region to the left of the dotted line) were defined as Constant_imm_genes, i.e., genes involved in the TCR-diversity-correlated pathways that are steadily expressed within the tumor mass. The prognostic value of the Constant_imm_genes was tested in 50 GBM patients (Fig. 5C–E) and in the published TCGA GBM dataset (Fig. 5F–H). As shown in Fig. 5C and F, the two cohorts of GBM patients were divided into two clusters according to the expression pattern of the Constant_imm_genes. The log-rank test results indicated a significant difference in prognosis between the two clusters. Figure 5D and G show the results of the overall survival analysis (p = 0.004 for our GBM cohort and p = 0.018 for TCGA cohort). Figure 5E and H showed the results of the disease-free survival analysis (p = 0.005 for our GBM cohort and p = 0.022 for the TCGA cohort).

## Discussion

Using TCR sequencing technology, some previous studies have revealed the spatial heterogeneity of TILs in ovarian cancers^9^, renal cell carcinomas^19^ and esophageal squamous cell carcinomas^20^. The largest difference between our method and the methods of these previous studies is in the starting material: we used RNA instead of genomic DNA. Using primer-specific reverse transcription, we focused on sequencing the RNA populations to facilitate the analysis of functionally rearranged TCRBs rather than non-functional TCRB allele rearrangements in the genomic DNA; the disadvantage of this approach is that unequal numbers of RNA molecules per cell might distort the detected TIL populations^37^. As the activation and expansion of T cell clones are strongly indicative of an ongoing immune response^38^, it is assumed that with careful control of PCR-bias, the RNA expression level of the T cell receptors can better reflect the functional status better than can DNA. To prevent the bias from being amplified, ARM-PCR^22^, which allows millions of highly similar sequences to be amplified in a semi-quantitative matter from a complex mixture, was applied. This method has been applied in many studies^16, 39, 40^.

The clonal expansion of TILs is caused by an epitope-specific T cell response. Theoretically, cancer rejection epitopes might be derived from two classes of antigens. A first class of potential cancer rejection antigens is formed by non-mutated proteins, to which T cell tolerance is incomplete. The other class is formed by so-called neoantigens, which are solely created by tumor-specific DNA alterations that result in the formation of novel protein sequences in many human tumors without a viral etiology^1^. Emerging data suggested that the recognition of such neoantigens is a major factor in the activity of clinical immunotherapies, which has stimulated a renewed interest in personalized therapies targeting cancer mutations^1–3^. In the present study, somatic mutations and TCRB clonality of ten tumor samples from two tumor masses of one patient were analyzed simultaneously. We found that although the multi-region samples from a single tumor shared more common mutations than did those from different tumors, the TIL populations from different regions of a single tumor mass did not share more common clones than those from different tumors. Moreover, we found that there was no significant correlation between TIL repertoire diversity and the number of non-synonymous mutations. Our results indicate that in the natural state of oncoimmunity, the main cause of lymphocyte activation and invasion of tumors might not be the neoantigens encoded by tumor-specific somatic mutations, which could help explain how cancers avoid immuno-rejection. The success of current tumor immunotherapy might be attributed to the modulation of the immune system to overcome barriers to re-recognize and react to such cancer neoantigens instead of simply reactivating the cytolytic capacity of the existing expanded T cell clones.

Repertoire diversity is a fundamental determinant of the competence of the immune system^16^. We found that the immune repertoire diversity of the GBM patient might be systemically depressed by the cancerous state, as an estimated 20% or more of the αβ-T cells in the peripheral blood might be tumor-related (although not tumor-rejected). With respect to the tumor microenvironment, TIL diversity showed a significant positive correlation with the activation of immunity-related pathways. In these pathways, we found a signature of 23 genes with expression levels were minimally affected by the intratumor heterogeneity, and we showed that their collective expression pattern was correlated with the patient prognosis at the population level. These results indicated that although the heterogeneity of intratumor immune status was non-negligible, the overall level of the immune response in GBM tissue was measurable and is potentially related to the clinical prognosis.

In this study, only one GBM patient was involved in the TIL and somatic mutation detection analysis. However, this patient, who had both a primary tumor and a related metastatic GBM lesion, was very appropriate for our study because both the TCR repertoire and the somatic mutations were patient-specific; the connection between these two features might be more assessable when using paired primary and metastatic tumor masses than when using tumors from different patients. Additional similar cases might offer additional information. Moreover, dividing of TILs into functional subtypes according to their surface markers could be conducted in future studies to enhance the analyses.

Despite study limitations, our research provides new insight into the heterogeneity of TILs in GBM through an integrated analysis of multi-omics data.

## Electronic supplementary material

Supplementary Information
