# Supplementary material for: Heterogeneity of tumor-infiltrating lymphocytes ascribed to local immune status rather than neoantigens by multi-omics analysis of glioblastoma multiforme
Source: Sci Rep. 2017 Jul 31;7:6968. doi: 10.1038/s41598-017-05538-z (PMC5537248; doi:10.1038/s41598-017-05538-z)
Supplement: Supplementary file 1 — Supplementary Information [file 41598_2017_5538_MOESM1_ESM.pdf]

Heterogeneity of tumor-infiltrating lymphocytes ascribed to local immune status rather than neoantigens by multi-omics analysis of glioblastoma multiforme

Lin Feng*, Haipeng Qian*, Xuexin Yu*, Kan Liu, Ting Xiao, Chengli Zhang, Manchao Kuang, Shujun Cheng, Xueji Li#, Jinghai Wan#, Kaitai Zhang#

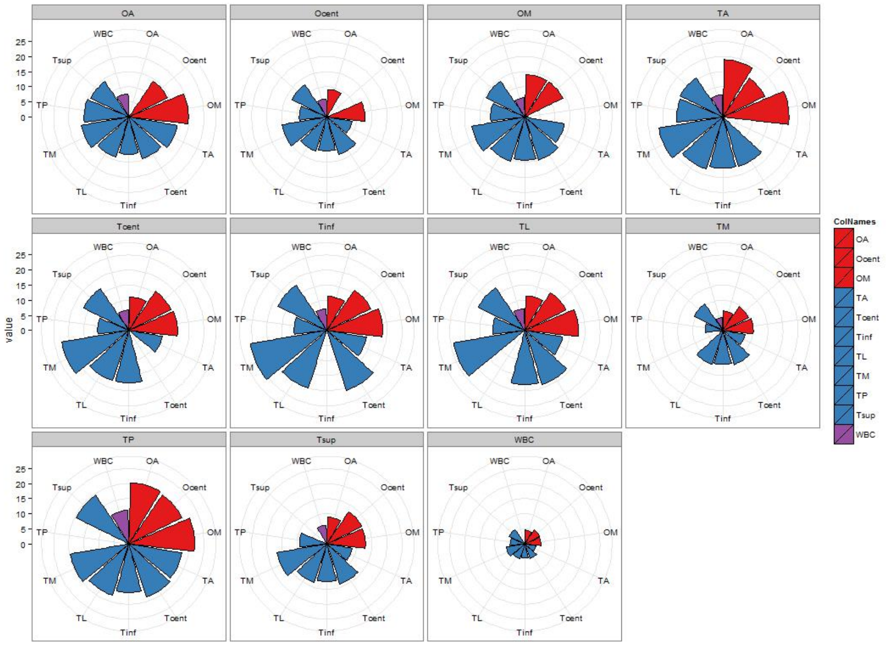

Figure S1: The overlap of the TCRB clones between pairwise samples. Each of the rose diagram displays the percentage of the overlapped TCRB clones between the sample named the panel (the sample marked in the grey bar of the panel) and the other samples (listed around the petals). The proportions of the overlap are represented by the length of petals, with red, blue and purple indicating the occipital, temporal tumor samples and peripheral blood, respectively.

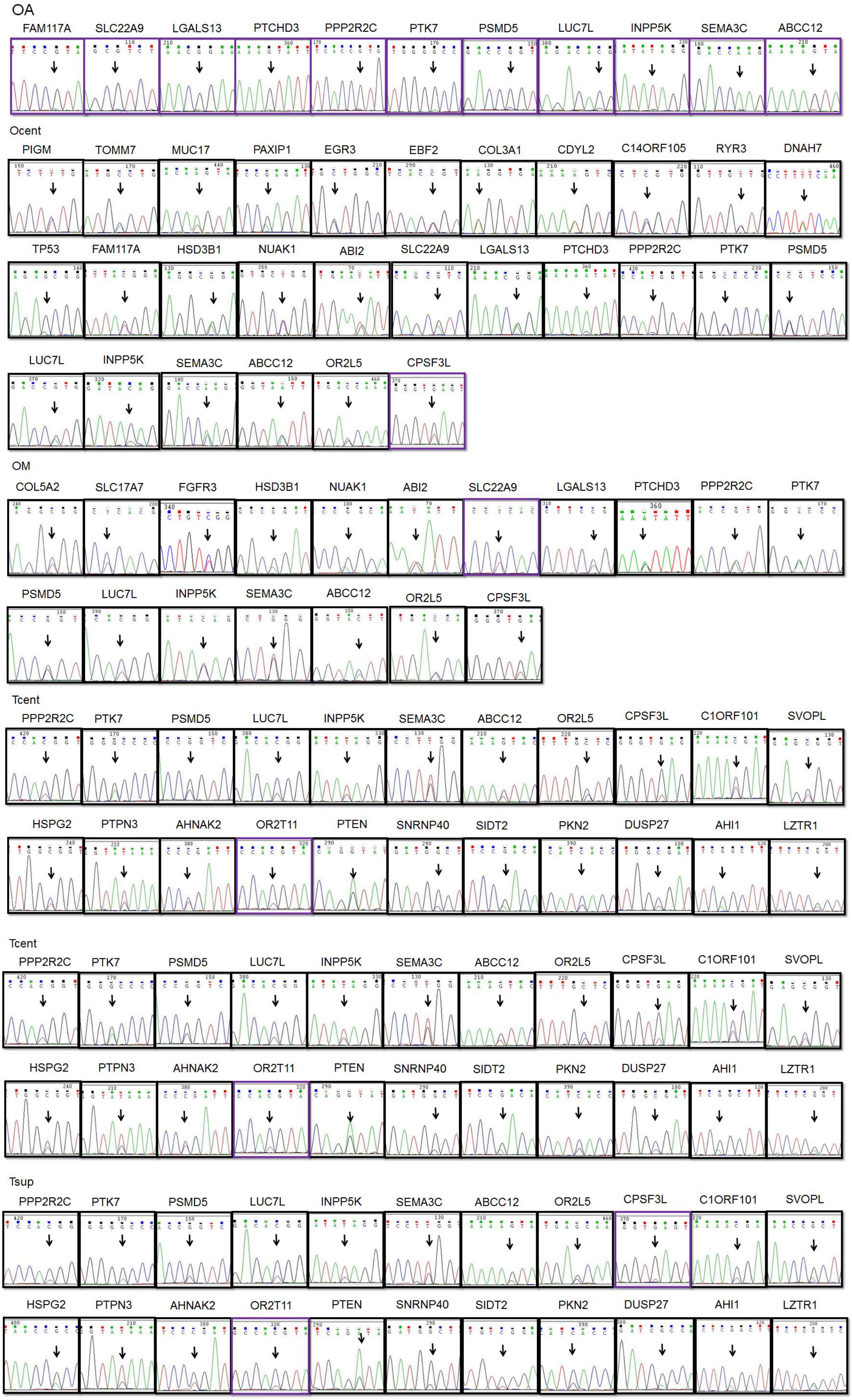
OA
FAM117A SLC22A9 LGALS13 PTCHD3 PPP2R2C PTK7 PSMD5 LUC7L INPP5K SEMA3C ABCC12
Ocent
PIGM TOMM7 MUC17 PAXIP1 EGR3 EBF2 COL3A1 CDYL2 C14ORF105 RYR3 DNAH7
TP53 FAM117A HSD3B1 NUAK1 ABI2 SLC22A9 LGALS13 PTCHD3 PPP2R2C PTK7 PSMD5
LUC7L INPP5K SEMA3C ABCC12 OR2L5 CPSF3L
OM
COL5A2 SLC17A7 FGFR3 HSD3B1 NUAK1 ABI2 SLC22A9 LGALS13 PTCHD3 PPP2R2C PTK7
PSMD5 LUC7L INPP5K SEMA3C ABCC12 OR2L5 CPSF3L
Tcent
PPP2R2C PTK7 PSMD5 LUC7L INPP5K SEMA3C ABCC12 OR2L5 CPSF3L C1ORF101 SVOPL
HSPG2 PTPN3 AHNAK2 OR2T11 PTEN SNRNP40 SIDT2 PKN2 DUSP27 AHI1 LZTR1
Tcent
PPP2R2C PTK7 PSMD5 LUC7L INPP5K SEMA3C ABCC12 OR2L5 CPSF3L C1ORF101 SVOPL
HSPG2 PTPN3 AHNAK2 OR2T11 PTEN SNRNP40 SIDT2 PKN2 DUSP27 AHI1 LZTR1
Tsup
PPP2R2C PTK7 PSMD5 LUC7L INPP5K SEMA3C ABCC12 OR2L5 CPSF3L C1ORF101 SVOPL
HSPG2 PTPN3 AHNAK2 OR2T11 PTEN SNRNP40 SIDT2 PKN2 DUSP27 AHI1 LZTR1

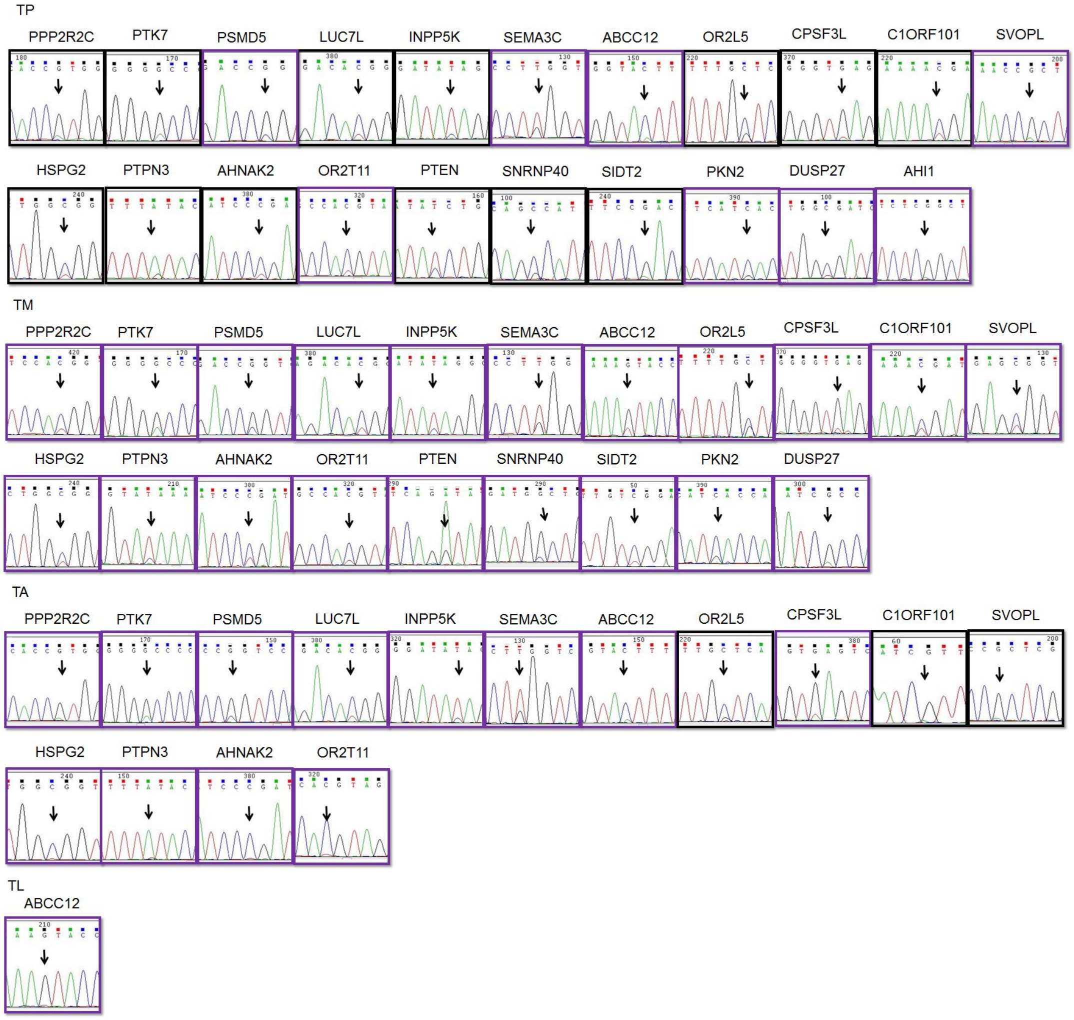

Figure S2: Sanger sequencing validation of mutations identified by exome sequencing in Patient 1. The figure shows the mutations in each regional tumor samples displayed on Figure 4A validated using Sanger sequencing. Mutations are indicated with an arrow. The color of the outlies indicate whether the mutations were discovered by NGS (black for yes, purple for no).

TableS1_Clinical_Information

Patient ID	Gender	Age	Overall survival (months)	Events (dead = 1)	Disease free survival (months)	Events (relapse = 1)	Cluster
Patient 1	M	51	25.00	0	25.00	1	NA
GBM01	F	49	17.29	1	9.14	1	red
GBM02	F	35	42.71	0	33.11	1	black
GBM03	M	46	12.53	1	6.64	1	red
GBM04	M	64	8.32	1	8.32	1	red
GBM05	F	35	13.58	1	10.42	1	red
GBM06	M	54	25.55	1	22.85	1	black
GBM07	M	32	46.26	1	37.87	1	black
GBM08	M	61	31.63	0	9.11	1	red
GBM09	F	42	4.47	1	1.45	1	red
GBM10	F	42	16.64	1	11.05	1	red
GBM11	M	56	18.21	1	15.09	1	red
GBM12	F	28	42.87	0	42.87	0	black
GBM13	M	55	12.59	1	8.12	1	red
GBM14	F	45	34.92	0	34.92	0	red
GBM15	M	59	16.34	1	11.24	1	black
GBM16	F	39	13.25	1	7.40	1	red
GBM17	M	56	6.51	1	3.55	1	red
GBM18	M	62	10.68	1	10.68	1	red
GBM19	F	44	33.67	1	23.47	1	red
GBM20	M	38	11.70	1	8.05	1	black
GBM21	M	37	27.16	1	27.16	1	red
GBM22	F	24	21.99	0	21.99	0	red
GBM23	M	37	7.99	1	7.99	1	red
GBM24	F	58	13.02	1	13.02	1	red
GBM25	F	38	35.80	1	30.77	1	black
GBM26	M	42	13.78	1	3.55	1	black
GBM27	NA	43	25.97	0	25.97	0	black
GBM28	F	35	19.20	1	19.20	1	red
GBM29	M	59	17.52	1	10.26	1	red
GBM30	F	60	17.42	1	9.93	1	black
GBM31	F	56	15.78	0	15.78	0	red
GBM32	F	45	9.24	1	3.02	1	red
GBM33	F	57	15.19	1	5.88	1	red
GBM34	M	18	42.44	0	42.44	0	black
GBM35	F	43	9.21	0	9.21	0	black
GBM36	F	40	18.51	1	12.85	1	red
GBM37	F	33	18.58	1	18.58	1	red

GBM38	F	57	11.93	1	11.93	1	red
GBM39	F	27	18.05	1	7.99	1	black
GBM40	M	47	19.50	1	12.99	1	black
GBM41	F	54	22.82	0	20.71	1	red
GBM42	M	40	6.18	1	3.25	1	red
GBM43	M	52	11.05	1	3.78	1	red
GBM44	F	43	19.43	1	16.96	1	red
GBM45	F	68	9.86	1	7.30	1	red
GBM46	M	28	21.44	0	21.44	0	black
GBM47	M	38	31.76	1	12.49	1	red
GBM48	M	52	11.11	1	2.14	1	red
GBM49	M	55	12.69	1	7.10	1	red
NA: not available							

TableS2_TCR_Sequencing_Primers

primer name	sequence
TRBV1Fo	AATGAAACGTGAGCATCTGG
TRBV2Fo	GTGTCCCCATCTCTAATCAC
TRBV3Fo	TATGTATTGGTATAAACAGG
TRBV4Fo	GTCTTTGAAATGTGAACAAC
TRBV5Fo	GATCAAAACGAGAGGACAGC
TRBV6aFo	GTGTGCCCAGGATATGAACC
TRBV6bFo	CAGGATATGAGACATAATGC
TRBV7Fo	CTCAGGTGTGATCCAATTTC
TRBV9Fo	GAGACCTCTCTGTGTACTGG
TRBV10Fo	GGAATCACCCAGAGCCCAAG
TRBV11Fo	CCTAAGGATCGATTTTCTGC
TRBV12Fo	AGGTGACAGAGATGGGACAA
TRBV13Fo	CTATCCTATCCCTAGACACG
TRBV14Fo	AGATGTGACCCAATTTCTGG
TRBV15Fo	TCAGACTTTGAACCATAACG
TRBV16Fo	TATTGTGCCCCAATAAAAGG
TRBV17Fo	ATCCATCTTCTGGTCACATG
TRBV18Fo	GCAGCCCAATGAAAGGACAC
TRBV19Fo	TGAACAGAATTTGAACCACG
TRBV20Fo	TCGAGTGCCGTTCCCTGGAC
TRBV21Fo	GCAAAGATGGATTGTGTTCC
TRBV23Fo	CATTTGGTCAAAGGAAAAGG
TRBV24Fo	ATGCTGGAATGTTCTCAGAC
TRBV25Fo	CTCTGGAATGTTCTCAAACC
TRBV26Fo	CCCAGAATATGAATCATGTT
TRBV27Fo	TTGTTCTCAGAATATGMCC
TRBV28Fo	ATGTGTCCAGGATATGGACC
TRBV29Fo	TCACCATGATGTTCTGGTAC
TRBV30Fo	TGTGGAGGGAACATCAAACC
TRBCRo	GTGTGGCCTTTTGGGTGTGG
TRBV1Fi	TCTTTCCCTACACGACGCTCTTCCGATCTCATTGAAAACAAGACTGTGC
TRBV2Fi	TCTTTCCCTACACGACGCTCTTCCGATCTTGAAATCTCAGAGAAGTCTG
TRBV3Fi	TCTTTCCCTACACGACGCTCTTCCGATCTCTCTAAGAAATTTCTGAAGA
TRBV4Fi	TCTTTCCCTACACGACGCTCTTCCGATCTGGAGCTCATGTTTGTCTACA
TRBV5aFi	TCTTTCCCTACACGACGCTCTTCCGATCTCAGGGGCCCCAGTTTATCTT
TRBV5bFi	TCTTTCCCTACACGACGCTCTTCCGATCTGAAACARAGGAAACTTCCCT
TRBV6aFi	TCTTTCCCTACACGACGCTCTTCCGATCTGGTATCGACAAGACCCAGGC
TRBV6bFi	TCTTTCCCTACACGACGCTCTTCCGATCTTAGACAAGATCTAGGACTGG
TRBV7aFi	TCTTTCCCTACACGACGCTCTTCCGATCTTCTAATTTACTTCCAAGGCA
TRBV7bFi	TCTTTCCCTACACGACGCTCTTCCGATCTTCCCAGAGTGATGCTCAACG

TRBV7CFi	TCTTTCCCTACACGACGCTCTTCCGATCTACTTACTTCAATTATGAAGC
TRBV7dFi	TCTTTCCCTACACGACGCTCTTCCGATCTCCAGAATGAAGCTCAACTAG
TRBV9Fi	TCTTTCCCTACACGACGCTCTTCCGATCTCTCATTCAGTATTATAATGG
TRBV10Fi	TCTTTCCCTACACGACGCTCTTCCGATCTGACATGGGCTGAGGCTGATC
TRBV11Fi	TCTTTCCCTACACGACGCTCTTCCGATCTACTCTCAAGATCCAGCCTGC
TRBV12aFi	TCTTTCCCTACACGACGCTCTTCCGATCTTGCAGGGACTGGAATTGCTG
TRBV12bFi	TCTTTCCCTACACGACGCTCTTCCGATCTGTACAGACAGACCATGATGC
TRBV13Fi	TCTTTCCCTACACGACGCTCTTCCGATCTAAGATGCAGAGCGATAAAGG
TRBV14Fi	TCTTTCCCTACACGACGCTCTTCCGATCTAGTCTAAACAGGATGAGTCC
TRBV15Fi	TCTTTCCCTACACGACGCTCTTCCGATCTAAAGATTTTAACAATGAAGC
TRBV16Fi	TCTTTCCCTACACGACGCTCTTCCGATCTAATGTCTTTGATGAAACAGG
TRBV17Fi	TCTTTCCCTACACGACGCTCTTCCGATCTAACATTGCAGTTGATTCAGG
TRBV18Fi	TCTTTCCCTACACGACGCTCTTCCGATCTAATATCATAGATGAGTCAGG
TRBV19Fi	TCTTTCCCTACACGACGCTCTTCCGATCTTTTCAGAAAGGAGATATAGC
TRBV20Fi	TCTTTCCCTACACGACGCTCTTCCGATCTGATGGCAACTTCCAATGAGG
TRBV21Fi	TCTTTCCCTACACGACGCTCTTCCGATCTCGCTGGAAGAAGAGCTCAAG
TRBV23Fi	TCTTTCCCTACACGACGCTCTTCCGATCTGAATGAACAAGTTCTTCAAG
TRBV24Fi	TCTTTCCCTACACGACGCTCTTCCGATCTGTCAAAGATATAAACAAAGG
TRBV25Fi	TCTTTCCCTACACGACGCTCTTCCGATCTTAATTCCACAGAGAAGGGAG
TRBV26Fi	TCTTTCCCTACACGACGCTCTTCCGATCTATTCACCTGGCACTGGGAGC
TRBV27Fi	TCTTTCCCTACACGACGCTCTTCCGATCTTGAGGTGACTGATAAGGGAG
TRBV28Fi	TCTTTCCCTACACGACGCTCTTCCGATCTAAAAGGAGATATTCCTGAGG
TRBV29Fi	TCTTTCCCTACACGACGCTCTTCCGATCTCTGGACAGAGCCTGACACTG
TRBV30Fi	TCTTTCCCTACACGACGCTCTTCCGATCTTTCTACTCCGTTGGTATTGG
TRBCRi	TTCTTAGCGTATTGGAGTCCTCTGATGGCTCAAACACAGC
SuperF	AATGATACGGCGACCACCGAGATCTACACTCTTTCCCTACACGACG
SuperR	CAAGCAGAAGACGGCATACGAGAT-barcode-GTGACTGGAGTTCAGACGTGTG

Table S3: TCR sequencing statistics in Patient 1.

Sample ID	TCRB total reads count	unique TCRB clones
OA	3,707,664	71,328
Ocent	4,709,566	112,211
OM	4,445,795	100,645
TA	2,657,495	61,145
Tcent	3,303,584	93,504
Tinf	2,754,626	77,886
TL	2,925,236	87,260
TM	4,469,758	177,554
TP	3,463,513	52,372
Tsup	4,953,931	112,264
Peripheral blood	3,549,276	119,210

Table S4. The percentage of TCRB clones with different frequency.

SID	TCRB frequency (%)					
	<0.0001%	0.0001-0.001%	0.001-0.01%	0.01-0.1%	0.1-1%	>1%
OA	72.071	22.067	4.594	1.090	0.138	0.040
Ocent	69.386	25.215	4.385	0.816	0.175	0.023
OM	68.730	25.772	4.233	1.055	0.189	0.020
TA	69.603	25.237	3.635	1.342	0.169	0.014
Tcent	68.282	26.524	3.828	1.163	0.194	0.009
Tinf	68.765	25.886	3.812	1.327	0.199	0.011
TL	70.130	24.752	3.538	1.408	0.162	0.010
TM	73.233	21.462	4.258	0.953	0.088	0.006
TP	71.477	23.997	3.346	0.977	0.175	0.028
Tsup	69.661	25.544	3.581	1.035	0.167	0.012
Peripheral blood	68.973	25.127	5.352	0.237	0.310	0.001

TableS5_WES_statistics

Sample ID	Total clean reads count	Fraction of uniquely mapped on target	Coverage of target region	Average sequencing depth on target	Fraction of target covered with at least 20x
OA	127,569,986	71.20%	99.90%	105.55	96.90%
Ocent	174,376,592	55.70%	99.90%	103.03	96.60%
OM	137,502,752	72.40%	99.90%	106.4	96.80%
TA	140,455,784	71.10%	99.80%	102.9	96.50%
Tcent	176,887,576	55.10%	99.90%	102.64	96.90%
Tinf	148,604,524	62.20%	99.90%	104.62	96.70%
TL	148,372,760	70.80%	99.80%	108.7	96.80%
TM	131,655,000	70.30%	99.80%	103.68	96.70%
TP	124,374,048	72.10%	99.90%	108.1	96.70%
Tsup	126,660,426	72.40%	99.80%	106.61	96.70%
Peripheral blood	131,577,926	71.50%	99.90%	106.18	96.80%

TableS6_Somatic_mutations

Gene	Chromosome	Position	Nucleotide Variant	Amino Acid Change
ABCC12	16	48162533	C>A	S451I
ABI2	2	204231745	C>A	S88Y
AHI1	6	135778798	G>A	R329X
AHNAK2	14	105420407	C>T	G461R
C14orf105	14	57949844	C>T	R111Q
C1orf101	1	244715979	G>A	V298I
CDYL2	16	80718599	T>A	K151M
COL3A1	2	189872252	A>T	K1094N
COL5A2	2	189904156	G>A	A1256V
CPSF3L	1	1254825	G>T	H100N
DNAH7	2	196825169	T>A	E902D
DUSP27	1	167086736	G>A	R126H
EBF2	8	25898455	C>T	G118S
EGR3	8	22548767	C>G	S90T
FAM117A	17	47788783	C>T	R399H
FGFR3	4	1808859	C>T	S652L
HSD3B1	1	120050160	C>T	R21C
HSPG2	1	22160001	C>T	R3646
INPP5K	17	1413029	T>C	Y33C
LGALS13	19	40095882	C>T	R53C
LUC7L	16	277296	C>T	R34H
LZTR1	22	21343948	C>T	R210X
MUC17	7	100676063	A>T	S456C
NUAK1	12	106461214	G>A	P451L
OR2L5	1	248185656	G>C	S136T
OR2T11	1	248790069	C>T	V121M
PAXIP1	7	154739692	C>T	R948Q
PIGM	1	160000598	C>T	R311K
PKN2	1	89298442	G>C	D860H
PPP2R2C	4	6377562	G>A	T144M
PSMD5	9	123580369	G>C	R444G
PTCHD3	10	27687377	G>A	T717I
PTEN	10	89653782	A>G	Y27C
PTK7	6	43096843	G>A	A70T
PTPN3	9	112216813	T>C	I111V
RYR3	15	34078139	A>G	N3182
SEMA3C	7	80374553	T>G	Q638P
SIDT2	11	117059519	G>A	D419N

SLC17A7	19	49934292	C>T	G457R
SLC22A9	11	63175593	C>T	T433M
SNRNP40	1	31754318	G>C	A186G
SVOPL	7	138329502	C>T	R98H
TOMM7	7	22857638	G>T	P45T
TP53	17	7577095	G>C	D149E
